# Trends in and Risk Factors for Bicycle-Related Mortality in an Ageing Cycling-Centric Country: Analysis of Japanese Administrative Data

**DOI:** 10.3390/ijerph22030322

**Published:** 2025-02-21

**Authors:** Sayo Tanaka, Keiki Shimizu, Stuart Gilmour

**Affiliations:** 1 Graduate School of Public Health, St. Luke’s International University, 3-6-2 Tsukiji, Chuo-ku, Tokyo 104-0045, Japan; sgilmour@slcn.ac.jp; 2 Department of Orthopedic Surgery, Ibaraki Welfare and Medical Center, 1872-1 Motoyoshida-cho, Mito-shi, Ibaraki 310-0836, Japan; 3 Trauma and Resuscitation Center, Tokyo Metropolitan Tama Medical Center, 2-8-29 Musashidai, Fuchu-shi, Tokyo 183-0042, Japan; icu240024@yahoo.co.jp

**Keywords:** cyclist, ageing society, road traffic injury, trend, risk factors

## Abstract

Japan has the most ageing population in the world with a high population of bicycle users, and the percentage of older cyclists continues to grow as the population ages. At the same time, the proportion of bicycle-related collisions is increasing. The aim of this study is to analyse trends and risk factors for bicycle injuries and deaths in Japan in order to suggest preventive measures, using data from vital statistics and the National Police Agency to calculate incidence rate ratios (IRR), age-standardised mortality rates, and annual percent changes, by ten-year-interval age groups. Data from the Japan Trauma Data Bank was analysed for demographic information about injuries. The risk of casualties was high in the younger generation and lower in the older population. However, the risk of mortality increased rapidly with age, with people over 70 years old facing more than 10 times the risk of younger age groups (IRR = 12.62). Casualty and mortality rates were declining in all age groups until the year 2020 (range: −9.77% to −4.95%, −8.61% to −1.07%, respectively). However, lethality of bicycle collisions showed no significant reduction. Current methods have not been effective in reducing bicycle-related lethality in Japan, especially for the older population, and should be improved to ensure that bicycle transportation is safe for all road users.

## 1. Introduction

Bicycles are a common mode of transportation method in many countries, with global bicycle ownership reported at 42% [1]. Japan is among the top ten largest bicycle users globally, with more than 72 million bicycle owners [2]. People living in urban areas have relied on bicycles as their transportation method, mainly because they allow people to travel freely and rapidly on narrow and crowded roads [3]. In the past decade, the Japanese government enacted the Bicycle Utilization Promotion Plan to promote more bicycle use to improve the environment, traffic control, transportation in times of disasters, healthy lifestyles, and also to provide transportation for older people [3,4].

However, while Japan’s bicycle culture allows widespread, unrestricted use of bicycles, there are several risks for bicycle injuries. First, the roads in urban areas are narrow and crowded, and do not separate lanes for cars, bicycles, and pedestrians, and the number of designated lanes for bicycles are limited [5]. Secondly, the law allows cyclists 16 years old or older to carry two young children under the age of six on one bike, a widespread practice among parents with children [6]. On the other hand, 63% of community-dwelling older adults in urban areas regularly ride bicycles [7]. The number of older cyclists is increasing, as 20% of older adults who returned their drivers’ licence began using bicycles as a mode of transportation [8]. At the same time, it has been reported that one in three older cyclists in urban areas sustain serious injuries that require hospital treatment [9,10]. Thirdly, there are no mandatory rules or laws for helmet use; instead, cyclists are asked only to make “efforts” [11]. This leaves unprotected and unstable bike riders roaming through road environments where there are many pedestrians, weaving between slow vehicular traffic and pedestrians, often while carrying small children. Since bicycles are a part of many Japanese people’s daily lives, there are other risks such as riding on a rainy day while holding an umbrella or using a cell phone while riding [12]. Some people intentionally use bicycles instead of cars when they go out drinking [12].

The police reported a total of 72,339 bicycle-related crashes in 2023, accounting for 23.5% of all road traffic crashes, which hit a record high [13]. In Tokyo, there were over 15,000 crashes involving cyclists in 2023, which was an increase of over 650 cases from 2022, and the proportion of bicycle-related crashes has increased from 36.1% to 46.3% over the past five years [13]. Among these crashes, injury rates were highest in people aged 15 to 19 years, but fatalities were concentrated in those aged 65 and older, with 60% of all bicycle-related deaths occurring in riders aged over 70 [14,15]. Since Japan is the world’s most ageing country with 29.1% of the population aged over 65 years in 2023, and this proportion is estimated to grow to 38.7% by 2070, it can be assumed that the percentage of injuries in this age group will increase [16]. Furthermore, it has been reported that over 70% of bicycle-related deaths in individuals under the age 65 and 65% in age 65 and older were associated with violations of traffic laws [13]. Since cyclists over 50 years old are increasing, it is likely that the number of collisions among the older generation due to law violations will also increase [2,17].

Although Japan has a substantial population of bicycle users, there is a limited body of research on bicycle-related injuries compared to other countries where bicycles are used on a daily basis [18]. Assessment of trends in and demographics of bicycle injuries is essential for prevention, to reflect on improvements in law, corporate education, road infrastructure, and insurance, as well as supporting a public health approach to understand the current situation of bicycle injuries in an ageing and bicycle-centric country such as Japan [19,20,21]. The aim of this study is to analyse trends in and risk factors for bicycle injuries and deaths in Japan by age group up to 80 years and older in order to suggest preventive measures based on the demographics of the high-risk population.

## 2. Materials and Methods

In order to assess the impact and trends from multiple perspectives, analysis was performed to identify the following indicators: (a) bicycle-related casualty per Japanese population, (b) bicycle-related mortality per Japanese population, (c) bicycle-related mortality per number of casualties, and (d) demographics of bike-related injuries compared with other vehicles. These include all casualties or mortalities in which cyclists were involved, including single-bicycle collisions.

### 2.1. Data Sources

Bicycle-related casualty data were obtained from the Annual Report of Traffic Accidents, released by the National Police Agency, which shows the number of annual casualties caused in traffic from 2010 to 2020 by vehicle type [22]. Mortality data were obtained from the vital statistics registration of the Ministry of Health, Labour, and Welfare in Japan, which provides a total of all deaths that occurred in Japan, based on the International Classification of Disease (ICD)-10 codes [23,24]. Codes v10-19 for pedal cycle riders injured in transport accidents between 1995 and 2020 were used for analysis. We classified age into ten-year intervals from ages 0 to 80 and over.

The Japan Trauma Data Bank (JTDB) database (year 2004–2018) was used for additional analysis on overall demographics of vehicle-related injuries in Japan. The JTDB was established in 2003, authorised by the Japanese Association for The Surgery of Trauma (JAST), and operated by Japan Trauma Care and Research (JTCR). In 2018, 280 emergency medical institutes across Japan participated in registering trauma cases to the JTDB [25]. Background information such as age, gender, and the occurrence of drinking and driving is available as aggregated data, as well as the injured body region, severity, and information on operation, to support the understanding of the situation of bicycle injuries in Japan. Severity was reported in five scales: Abbreviated Injury Scale (AIS), Injury Severity Score (ISS), Revised Trauma Score (RTS), Trauma and Injury Severity Score (TRISS), and Glasgow Coma Scale (GCS), to assess both anatomical and physiological scores. No personal identifiers were recorded or used, and ethical approval was not required based on Ethics Guidelines.

### 2.2. Statistical Analysis

Poisson regression analysis was performed to estimate incidence rate ratios (IRR) in bicycle-related casualty and mortality rates by year and age group, with ages 40–49 as the reference, as this age group includes the average age of overall bicycle-related injuries. An interaction term for year and age group was included in the analysis to determine age-specific trends in IRR. IRR was selected as a choice of method to show the significance of relative incidence rate in bike injuries by age group in comparison with the average. IRR was calculated as the exponential of the corresponding coefficient of the Poisson regression model, following the method of McCullagh and Nelder [26]. Linear combinations of year, age group category, and their interaction were calculated in order to estimate the modelled casualty and death rate, and presented as annual percent changes (APC). Casualty rates were analysed by population. Mortality rates were analysed separately using two denominators: (a) the Japanese population and (b) the number of bicycle-related casualties. Mortality rates were analysed separately by sex for analysis among the Japanese population. IRRs and APCs were considered to be statistically significant when they were different from zero with a *p*-value of <0.05. For analysis of overall trends, crude rates and directly age-standardised (DSR) casualty and mortality rates per 100,000 population were computed with the 2010 population of Japan as a reference [27]. DSR express the weighted average of casualty and mortality with the weights being equal to the proportion of people in each age group in a chosen standard population [28]. The numbers of casualties in 2010 were used as reference to calculate age-standardised mortality rates per 100,000 casualties.

For demographic data, proportions of bicycle-related factors were compared with car and motorcycle crashes using a chi-squared test or a logistic regression model. All variables included both drivers and passengers, except for drinking under the influence, in which only drivers were included, to assess the influence of drunk riders. All analyses were conducted in Stata/BE version 17.0 (Stata Corp LP, College Station, TX, USA).

## 3. Results

### 3.1. Incidence and Trends in Bicycle-Related Casualties

The number of bicycle-related casualties decreased from 151,676 in 2010 to 66,137 in 2020. Table 1 shows the IRR of bicycle-related casualties in Japan by age group from 2010 to 2020. The upper half of the table displays the results of IRRs and 95% confidence intervals (CI).

All values showed statistical significance. The average rate of bicycle-related casualties in ages 10–19 was 3.77 times the incidence of ages 40–49, but the rate decreased by more than half in age groups 20–29 (IRR = 1.66, 95% CI [1.64–1.68]), nearly a quarter in 30–39-year-olds (0.99 [0.79–1.00]), and ranged from 0.81 to 1.19 in ages 50–79. Casualty per population was the lowest in age 80 and over, with an IRR of 0.64 [95% CI, 0.63–0.65] times the risk of 40–49-year-olds. The lower half of the table shows the effect of the interaction of age and year on IRRs. The average rate of incidence of bicycle-related casualty declined in 0–9-year-olds, but these rates of decline were attenuated in ages 30–39, 50–59, and 80 or older. Table S1 in the Supplementary Materials shows the IRR of bicycle-related casualties in Japan by age group in years 2010 and 2020 to see possible differences by year and population demographics. Similar to overall results from 2010 to 2020, ages 10–19 had the highest incidence rate. However, the lowest incidence rate was in ages 0–9 instead of age 80 and over in year 2020.

**Figure 1:**
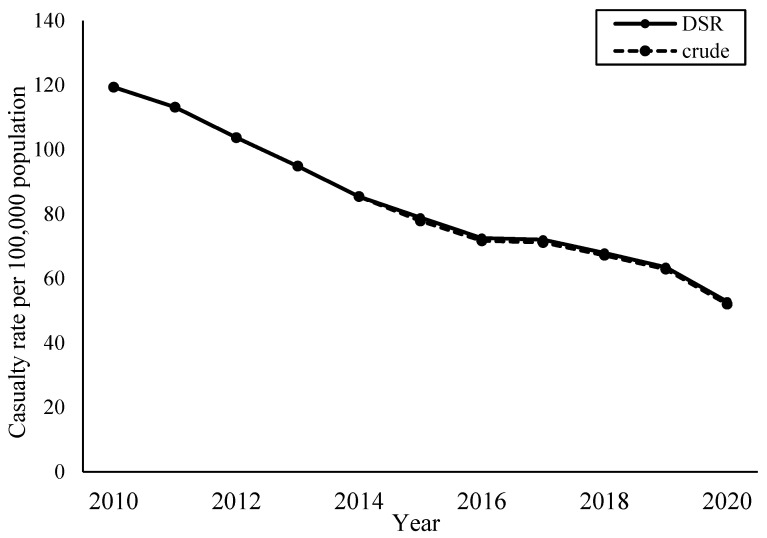
Crude and directly age-standardised casualty rates per 100,000 population (2010–2020).

Figure 1 shows the crude and DSR of bicycle casualties per 100,000 population from 2010 to 2020. Decreasing trends in casualties were observed in both crude and age-standardised rates throughout the observation period.

Table 2 shows the APCs in bicycle-related casualty per population by age group from 2010 to 2020, composed from the linear combinations of coefficients in the interaction model in Table 1. All APCs were statistically significant, where all results showed decreasing trends. The greatest decrease was seen in ages 0–9 with an APC of −9.77% [95% CI, −10.02 to −9.51] and the slowest decline was observed in the 80 and older population with a value of −4.95% [−5.22 to −4.68]. The trend in proportion of casualties by age group is shown as Supplemental Material (Figure S1).

### 3.2. Incidence and Trends in Bicycle-Related Mortality

#### 3.2.1. Mortality per Population

There were 2014 bicycle-related deaths in the year 1995 (1366 men and 648 women), and 523 (337 men, 186 women) in 2020.

Table 3 shows the IRR of bicycle-related mortality per capita in Japan from 1995 to 2020 by gender. The IRR of bicycle-related mortality among boys aged 10–19 was 1.44 times that of those aged 40–49 years, but the rate decreased to just over half in age groups 20–29 (IRR = 0.42, 95% CI [0.36–0.50]) and 30–39 (0.50 [0.43–0.60]), and increased by age group after ages 40–49 years old. The IRR sharply increased to 12.62 [95% CI, 11.35–14.04] in men ages 70–79 and 28.15 [25.29–31.35] in ages 80 and over. For girls/women, analysis showed a similar trend to boys/men, except for ages 80 and over. The average rate of bicycle-related mortality for girls aged 10–19 was 1.57 times the mortality of ages 40–49. The rate increased by age group after ages 50–59 years old and was higher than that of men in 50- to 69-year-olds. The highest IRR for women was observed in age 70–79 at 10.67 [95% CI, 9.23–12.34] but decreased to 4.00 [3.36–4.75] in ages 80 and over. The average rate of incidence of bicycle-related mortality by year has declined in both genders, but these rates of declines were significantly attenuated in women and men aged 20–29 and women aged 80 and older. Table S2 shows Table 3 in separate periods, from 1995–2009 and 2010–2020 for comparable purposes. Both time periods showed similar results to Table 3. However, women older than 60 years old showed significantly higher risk of death in the previous decade.

**Figure 2:**
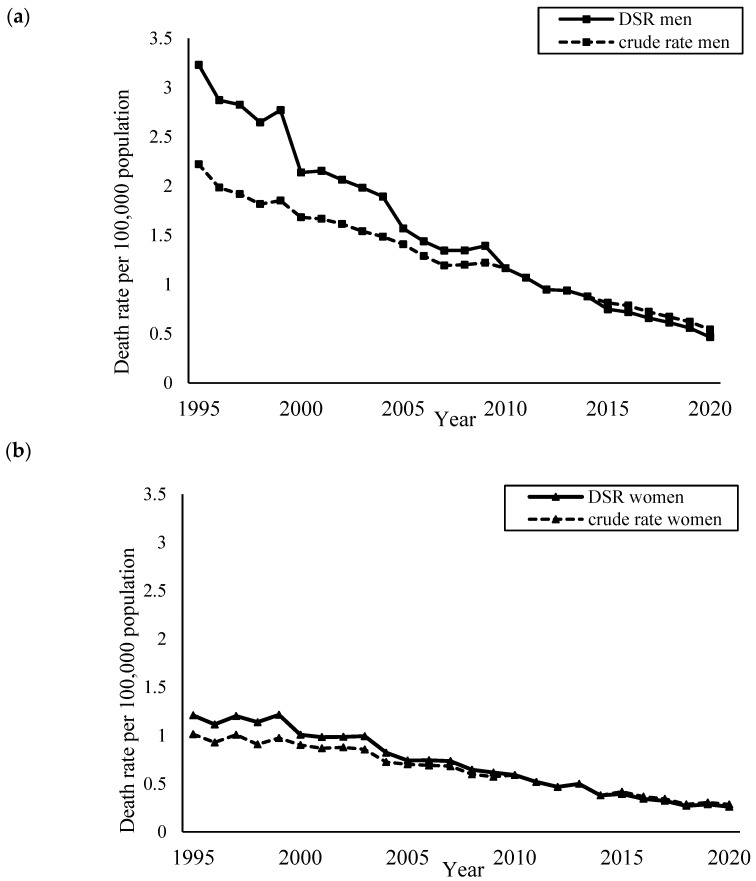
Crude and directly age-standardised mortality rates per 100,000 population among (**a**) men and (**b**) women (1995–2020).

Figure 2 shows the crude and age-standardised mortality rates per 100,000 population by gender, from 1995 to 2020. Men had a higher rate of deaths than women throughout the observed period. Decreasing trends were observed in both crude and age-standardised rates for both genders throughout those periods with larger reductions in mortality among men.

All annual percentage changes (APCs) were statistically significant for both genders, and all results showed decreasing trends (Table 4). For boys/men, the greatest decrease was seen in ages 0–9 with an APC of −8.54% [95% CI, −9.84 to −7.22] and the slowest decrease was observed in men in their 20s (−1.07% [−3.16 to −0.97]). Women showed similar trends to men until their 50s, with a large APC of −6.96% [95% CI, −9.13 to −4.74] in ages 0–9. However, the greatest decrease was observed in women in their 50s (IRR = −8.61, 95% CI [−9.37 to −7.86]), and the amount of decrease reduced with age. The APC for women aged 80 and older was −2.49% [95% CI, −3.19 to −1.78]. The trend in proportion of death by age group is shown as Supporting Information (Figure S2).

#### 3.2.2. Mortality Share of Casualties

Table 5 shows the IRR of bicycle-related mortality per number of casualties by age group from 2010 to 2020. The average rate of deaths over the number of casualties was significantly lower among cyclists aged 39 years and younger relative to 40–49-year-olds and decreased with age. In contrast, rates were significantly higher among those aged 50 and older and increased with age. The average death rate was highest in ages 80 years and older, which was 15.18 [95% CI, 12.72–18.11] times the age group of 40–49-year-olds, and lowest in ages 0–9-year-olds (0.39 [0.27–0.58]). Deaths per casualty did not show significant annual trends except for age nine and under with an upward trend.

**Figure 3:**
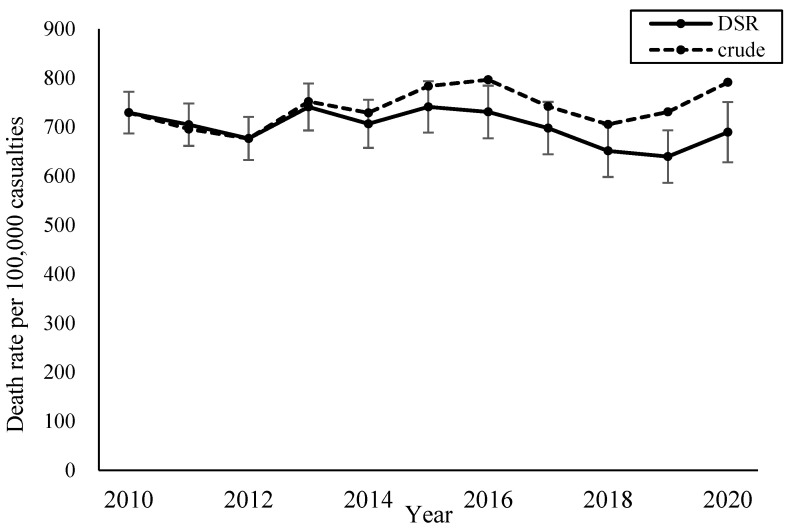
Crude and directly age-standardised mortality rates per 100,000 bicycle-related casualties (2010–2020).

Crude and age-standardised mortality rates per 100,000 casualties from 2010 to 2020 are shown in Figure 3. The crude mortality rate overall showed an increasing trend, but by adjusting with the age distribution of casualties in 2010, it showed a slightly decreasing trend.

Table 6 shows the APCs of bicycle-related mortality per casualty from 2010 to 2020. No significant change was seen for APCs, except for a 1.75% [95% CI, −3.02 to −0.47] decrease in the 80 years and older age group. Figure S3 shows that the proportion of deaths by age group has been stable throughout the ten years for the majority of age groups.

#### 3.2.3. Demographics and Severity of Bicycle-Related Injuries

Demographic data on vehicle-related injuries are shown in Table 7. Bicycle injuries had the highest percentage of drivers who reported drinking. They also experienced the highest percentage of head injuries (54.06%), which was more than twice as many compared to those who were injured in cars (26.23%), and operations on the head were also performed more commonly on bicycle riders (5.85%). The percentage of head injuries were highest at age 10–19, followed by age 70–79 and 80 and older (Table S3). All severity scores were most critical for cyclists among the three vehicles, except for ISS. The percentage of deaths was the highest at 9.62%, compared to 8.37% for cars, and 7.39% for motorcycles.

## 4. Discussion

This study shows that while the number and rate of bicycle-related crashes and deaths per 100,000 population have declined up to 2020, by analysing by age groups up to 80 years and older, it is notable that almost all age groups showed no significant decline in the risk of death per injury throughout the years, which indicates that the survival rate for bike-related injuries has not improved in the past decade. The older generation had the highest risk of bicycle-related deaths, and although the incidence of death is low in 20–39-year-olds, the mortality rate had minimal improvement compared to other groups. Compared to road injuries caused by other vehicles, cyclists were more prone to head injuries, more severely injured, and most likely to die in case of injury. They were also the most likely to drink and ride.

### 4.1. Current Situations in the High-Risk Groups and Age Groups with Minimal Decreasing Trend

In order to prevent road traffic injuries in the older generation, the Japanese government has promoted the return of licences by older drivers and encouraged them to use other transportation methods [29]. As a result, the many older people who forgo their licences are using bicycles as alternatives to maintain mobility [30,31]. The proportion of bicycle owners in these age groups increased from 21.3% in 2018 to 29.5% in 2021 [17]. However, bicycling is an accessible mode of transportation that comes with a high risk of failure, and it is a source of serious injury among older adults, which leads to an increase in injuries [7]. Our analysis showed that men over 70 years old and women aged 60–79 years old are at high risk of fatal injuries. Many older drivers think they are safe after switching to cycling after returning their drivers’ licence, but in reality, there are many factors among older people which lead to high risk in bike-related crashes in this group [10,32,33]. Furthermore, past studies have shown that older adults who were assessed to have a high likelihood of falling rode a bicycle regularly [7].

One significant result is the discrepancy of IRR between casualty and death (0.64 and 15.18) in the oldest people (age 80 and older). Past studies from other countries have shown that cyclists over 50 years old are more likely to be injured, but our study indicates that the oldest elderly people have a low risk of being involved in bicycle-related injuries compared to younger age groups [34]. However, despite the low risk of injury, they still have the highest death risk in case of injury, which emphasises the necessity for new measures in this growing age group. Further evaluation analysing the underlying factors, specifically in this age group, is urgently needed. However, statistics on exposure data of bicycle injuries are not reported specifically for this age group, which prevents further understanding.

On the other hand, cyclists aged 20–39 showed the least improvement in bicycle-related mortality over the years. In 2023, out of 72,339 bicycle-related collisions, 55,682 were caused by law violations [13]. Among those, people aged 24 and under violated the law the most, hence resulting in the large IRR of casualties in this age group [14]. In Japan, the main reason for using bicycles in the young adult population besides shopping and commuting, is to carry children [33]. Since 2009, the government legalised cyclists aged 16 and older to carry up to two children [11]. A possible reason for the limited decreasing trend in mortality rates in this age group may be the lack of compliance with bicycle safety and rules including parents carrying children [12]. Additionally, the media has reported more commercial bike-involved crashes after the outbreak of the COVID−19 pandemic, possibly due to more use of food delivery services [35]. Unfortunately, other than these facts, there is limited research on the causes of bicycle injuries in this age group [13,14,36,37,38].

### 4.2. Current Efforts by Japanese Society to Reduce Bicycle-Related Injuries

The Japanese government has proposed several policies to promote safety among cyclists from environmental, vehicle, and educational aspects. For example, Japanese electric bikes (e-bikes), for which ownership has more than doubled from 2012 to 2021, are required to be manufactured such that the assist ratio decreases after 10 km/h, and to avoid speeding, motor assist is more strictly restricted in Japan compared to other countries [17,39,40]. The government has also encouraged prefectures to obligate insurance coverage for bicycle owners, and finally, the obligation for cyclists in all age groups to make efforts to wear helmets, which started in April, 2023 [11,41]. Although it is not stated as ‘mandatory’, it is a step towards reducing fatal bicycle-related injuries. Finally, in November 2024, the Road Traffic Law was amended to strengthen penalties for driving under the influence of alcohol and while using a cell phone [42,43].

### 4.3. Suggestions for Reform to Improve Bicycle Safety in Japan

From our analysis and the past literature, it can be presumed that governmental efforts have shown success in decreasing injury and death among the Japanese population [44]. However, our study shows that specific measures are still needed especially to reduce fatal injuries in older adults and crashes in younger adults. Based on our results and past reports, we suggest mandatory helmet use, especially by older adults, and re-education of bicycle rules for both young and older adults.

Head injuries were the most affected body region for bicycle-related injuries in all age groups in our study and past reports [14,45,46]. Helmet use has been associated with reduced odds of head injuries among cyclists in other countries, and since the risk of death among casualties showed no improvement in our study, this should not be an exception in Japan [47,48,49,50]. Therefore, mandatory helmet use should be considered for all cyclists; especially for the older riders who have handed in their driving licence, to reduce the risk of mortality [47]. At the same time, 65 to 70% of fatal bicycle-injuries were caused by violating the law regardless of age [13]. Currently, educational events for cycling are mostly held for children in school, whereas adults have very limited opportunities to review or learn about bicycle safety [51]. Given that parents in their 20s’ and 30s’ tend to carry their children using bikes, educational events for this age group could be effective in reducing injuries among parents and also for bicycle-related death in 0–9year-olds who are carried by adults [22,52,53].

The strength of our study is the specific classification for older age groups. Ten-year-interval age groups are classified up to age 80, while most past studies on cyclists have classified the older population as ages 65 and older. Given Japan’s rapidly ageing population, more relevancies can be assessed not just for epidemiological purposes but also for policy change, when this generation is classified into more specific age groups. On the other hand, no covariates other than age and gender were used for adjustment, because of the limited information which could be derived from publicly available aggravated data. Despite the fact that Japan has the world’s most aging population, the majority of exposure factors on bicycle accidents in the older generation at a national level are reported as aged 65 and older as a whole, which is not in line with the reality of Japan’s current demographics and therefore of minimal use in developing preventive measures. Inclusion of exposure factors such as distances cycled, occupational, and other demographic information and analysis such as crash severity models may be relevant to understanding patterns of mortality in further studies.

## 5. Conclusions

While the Japanese government encourages older drivers to return their licences, based on the high IRR in deaths of the older cyclists, promotion of bike use in an ageing society may lead to increase in fatal injuries. This study showed that bicycle-related mortality has not decreased per casualty in Japan, which indicates the need for more comprehensive safety measures. These should include mandatory helmet use and re-education of cycling rules, especially for people aged over 70, to ensure a safe transition from cars to bicycle transportation for safety in all road users.

Efforts to improve safety should be implemented at various levels of society. The government should mandate helmet use, considering Japan’s status as the world’s most aged society. Education should target not only to the older generation but also police officers, families, and community organisations that serve elderly people. Older individuals need opportunities to become aware of the risks associated with cycling and to revisit bicycle laws, for example by being lectured when returning their drivers’ licences. Additionally, educating them on how their physical imbalance can increase the likelihood of falls is crucial and can also affect others. Families and communities also should be encouraged to be educated to recognise these risks and actively support and guide older individuals in choosing safer transportation options. Finally, local police need to be reminded of the heightened risks faced by the aging society, particularly as cycling becomes more common among older people, and implement strategies to prevent crashes through proper management and awareness.

As the Japanese population ages, and as the proportion of bicycle-related injuries increases, it is also important to develop credible indicators of the situation among older people, to evaluate environmental and vehicle-related factors in crashes, to suggest safety measures for this population. Re-evaluation of characteristics, strategies, and focus on implementation and country action based on population demographics, by re-examining the method in reporting bicycle injuries so more exposure factors can be in consideration, is key to achieving reductions in road traffic injuries for the next decade and beyond for all generations.

## Figures and Tables

**Table 1:** Results of Poisson regression analysis of incidence rate ratios of bicycle-related casualties per population by age group (2010–2020).

	Casualty				
	N	IRR	95% CI		*p*-Value
	(1,163,017)		Lower	Upper	
Year		0.93	0.93	0.94	<0.01
Age group (years)					
0–9	53,164	0.77	0.76	0.79	<0.01
10–19	309,028	3.77	3.73	3.81	<0.01
20–29	159,664	1.66	1.64	1.68	<0.01
30–39	130,941	0.99	0.79	1.00	<0.05
40–49	130,115	1 (reference)			
50–59	103,319	0.81	0.80	0.83	<0.01
60–69	118,851	1.01	1.00	1.03	<0.05
70–79	111,356	1.19	1.17	1.20	<0.01
80+	46,579	0.64	0.63	0.65	<0.01
Age group/year interaction					
0–9		0.97	0.96	0.97	<0.01
10–19		0.99	0.98	0.99	<0.01
20–29		1.00	0.99	1.00	<0.01
30–39		1.01	1.01	1.02	<0.01
40–49		1 (reference)			
50–59		1.02	1.01	1.02	<0.01
60–69		0.97	0.97	0.97	0.55
70–79		0.99	0.98	0.99	<0.01
80+		1.02	1.02	1.02	<0.01

CI: confidence interval, IRR: incidence rate ratio.

**Table 2:** Annual percent change in bicycle-related casualty per population among age groups (2010–2020).

	Casualty per Population			
Age Group (Years)	Annual % Change	95% CI		*p*-Value
		Upper	Lower	
0–9	−9.77	−10.02	−9.51	<0.01
10–19	−8.14	−8.24	−8.03	<0.01
20–29	−7.05	−7.20	−6.90	<0.01
30–39	−5.47	−5.63	−5.30	<0.01
40–49	−6.69	−6.86	−6.53	<0.01
50–59	−5.20	−5.39	−5.02	<0.01
60–69	−9.67	−9.84	−9.51	<0.01
70–79	−8.07	−8.25	−7.90	<0.01
80+	−4.95	−5.22	−4.68	<0.01

CI: confidence interval.

**Table 3:** Results of Poisson regression analysis of IRR of bicycle-related deaths per population by age group (1995–2020).

	Boys/Men				Girls/Women			
	IRR	95% CI		*p*-Value	IRR	95% CI		*p*-Value
		Lower	Upper			Lower	Upper	
Year	0.95	0.94	0.96	<0.01	0.94	0.93	0.95	<0.01
Age group (years)								
0–9	0.83	0.70	0.98	0.03	0.47	0.35	0.63	<0.01
19–10	1.44	1.26	1.64	<0.01	1.57	1.31	1.89	<0.01
20–29	0.42	0.36	0.50	<0.01	0.53	0.42	0.66	<0.01
30–39	0.50	0.43	0.60	<0.01	0.41	0.32	0.53	<0.01
40–49	1 (reference)				1 (reference)			
50–59	2.41	2.14	2.71	<0.01	3.38	2.88	3.95	<0.01
60–69	4.86	4.35	5.42	<0.01	6.21	5.35	7.20	<0.01
70–79	12.62	11.35	14.04	<0.01	10.67	9.23	12.34	<0.01
80+	28.15	25.29	31.35	<0.01	4.00	3.36	4.75	<0.01
Age group/year interaction								
0–9	0.96	0.95	0.98	<0.01	1.00	0.97	1.02	0.49
19–10	1.00	0.99	1.01	0.89	1.00	0.98	1.02	0.83
20–29	1.03	1.02	1.05	<0.01	1.04	1.02	1.06	<0.01
30–39	1.02	1.00	1.03	0.03	1.03	1.01	1.05	0.02
40–49	1 (reference)				1 (reference)			
50–59	0.99	0.98	1.00	0.06	0.96	0.95	0.98	<0.01
60–69	0.98	0.97	0.99	<0.01	0.98	0.97	0.99	<0.01
70–79	0.97	0.96	0.98	<0.01	0.99	0.98	1.00	0.05
80+	0.97	0.96	0.98	<0.01	1.03	1.01	1.04	<0.01

CI: confidence interval, IRR: incidence rate ratio.

**Table 4:** Annual percent change in bicycle-related death per population by gender among age groups (1995–2020).

Death per Population								
Boys/Men					Girls/Women			
Age Group (Years)	Annual% Change	95% CI		*p*-Value	Annual% Change	95% CI		*p*-Value
		Upper	Lower			Upper	Lower	
0–9	−8.54	−9.84	−7.22	<0.01	−6.96	−9.13	−4.74	<0.01
10–19	−5.11	−5.85	−4.36	<0.01	−5.80	−6.84	−4.75	<0.01
20–29	−1.07	−3.16	−0.97	<0.01	−2.61	−4.00	−1.21	<0.01
30–39	−3.56	−4.61	−2.50	<0.01	−4.36	−5.94	−2.74	<0.01
40–49	−5.03	−5.79	−4.27	<0.01	−6.29	−7.40	−5.17	<0.01
50–59	−6.36	−6.93	−5.80	<0.01	−8.61	−9.37	−7.86	<0.01
60–69	−6.67	−7.08	−6.25	<0.01	−7.49	−8.00	−6.97	<0.01
70–79	−7.74	−8.10	−7.40	<0.01	−6.42	−6.85	−5.99	<0.01
80+	−7.72	−8.06	−7.38	<0.01	−2.49	−3.19	−1.78	<0.01

CI: confidence interval.

**Table 5:** Results of Poisson regression analysis of incidence rate ratios of bicycle-related deaths per casualty by age group (2010–2020).

	Death				
	N	IRR	95% CI		*p*-Value
	(8254)		Lower	Upper	
Year		0.98	0.95	1.01	0.21
Age group (years)					
0–9	94	0.39	0.27	0.58	<0.01
10–19	406	0.44	0.35	0.55	<0.01
20–29	281	0.50	0.39	0.64	<0.01
30–39	291	0.66	0.51	0.85	<0.01
40–49	427	1 (reference)			
50–59	694	2.25	1.84	2.75	<0.01
60–69	1518	3.60	3.00	4.31	<0.01
70–79	2379	6.63	5.57	7.89	<0.01
80+	2164	15.18	12.72	18.11	<0.01
Age group/year interaction					
0–9		1.08	1.00	1.16	0.04
10–19		0.98	0.93	1.02	0.29
20–29		0.95	0.97	1.07	0.48
30–39		0.91	0.96	1.06	0.83
40–49		1 (reference)			
50–59		0.93	0.94	1.02	0.25
60–69		0.96	0.98	1.05	0.31
70–79		0.94	0.96	1.03	0.77
80+		0.93	0.95	1.02	0.42

CI: confidence interval, IRR: incidence rate ratio.

**Table 6:** Annual percent change in bicycle-related death per casualty among age groups (2010–2020).

	Death per Casualty			

Age Group (Years)	Annual% Change	95% CI		*p*-Value
		Upper	Lower	
0–9	+5.65	−0.98	+12.72	0.10
10–19	−3.08	−6.11	−0.05	0.05
20–29	−0.04	−3.74	+3.80	0.98
30–39	−3.63	−7.27	+0.14	0.06
40–49	−2.29	−5.25	+0.77	0.14
50–59	−2.28	−4.51	−0.00	0.05
60–69	−1.39	−0.23	+3.03	0.09
70–79	−0.30	−0.15	+0.95	0.63
80+	−1.75	−3.02	−0.47	<0.01

CI: confidence interval.

**Table 7:** Overall demographics of traffic-related injuries by vehicle (2004–2018).

	Overall ^a^ N = 126,338		Bicycle N = 25,092		Car N = 37,401 (Driver N = 27,823)		Motorcycle N = 36,695 (Driver N = 35,308)	
	N	(%)	N	(%)	N	(%)	N	(%)
**Age(mean(SD))**	47.72	(23.49)	48.75	(25.59)	**48.78**	**(21.84)**	40.65	(19.42)
**Gender**								
**Boys/Men**	85,779	(67.90)	15,619	(62.25)	24,229	(64.78)	**30,918**	**(84.26)**
**Girls/Women**	40,529	(32.08)	9466	(37.73)	13,159	(35.18)	5772	(15.73)
**Drinking ^b^**	9324	(7.38)	**2111**	**(8.41)**	2045	(7.35)	1805	(8.15)
**Region**								
**Head**	49,309	(40.49)	**13,127**	**(54.06)**	9409	(26.23)	12,319	(34.82)
**Face**	6442	(5.29)	1127	(4.64)	1865	(5.20)	**2321**	**(6.56)**
**Neck**	349	(0.29)	35	(0.14)	**170**	**(0.47)**	107	(0.30)
**Thorax**	23,421	(19.23)	2378	(9.79)	**10,259**	**(28.60)**	7195	(20.34)
**Abdomen**	5145	(4.22)	685	(2.82)	**2687**	**(7.49)**	1257	(3.55)
**Spine**	15,383	(12.63)	2506	(10.32)	**6698**	**(18.67)**	3868	(10.93)
**Upper Extremity**	5966	(4.90)	1314	(5.41)	1433	(3.99)	**2423**	**(6.85)**
**Lower Extremity**	14,758	(12.12)	2978	(12.26)	2907	(8.10)	**5600**	**(15.83)**
**Severity Scores**								
**AIS (M(SD))**	2.59	(1.05)	**2.66**	**(0.99)**	2.48	(1.10)	2.56	(1.03)
**ISS (M(SD))**	17.63	(13.41)	16.65	(11.89)	15.96	(13.19)	**17.16**	**(12.67)**
**RTS (M(SD))**	6.99	(1.89)	**7.09**	**(1.65)**	7.13	(1.79)	7.15	(1.72)
**TRISS (M(SD))**	0.86	(0.26)	**0.87**	**(0.24)**	0.88	(0.24)	0.90	(0.23)
**GCS (M(SD))**	12.69	(3.88)	**12.69**	**(3.78)**	13.20	(3.52)	13.09	(3.58)
**No operation**	53,220	(42.13)	9444	(37.64)	14,435	(38.60)	**17,576**	**(47.90)**
**Operation type**								
**Head**	4214	(3.34)	**1458**	**(5.85)**	493	(1.32)	958	(2.61)
**Thoracic**	2260	(1.79)	264	(1.05)	**691**	**(1.85)**	648	(1.77)
**Abdominal**	4771	(3.80)	333	(1.33)	**2764**	**(7.36)**	1005	(2.74)
**Fracture**	26,573	(21.04)	4519	(18.01)	6322	(16.90)	**10,492**	**(28.59)**
**Angioplasty**	318	(0.25)	22	(0.09)	85	(0.23)	150	(0.41)
**Death**	13,487	(10.68)	**2413**	**(9.62)**	3132	(8.37)	2712	(7.39)

AIS: Abbreviated Injury Scale, GCS: Glasgow Coma Scale, ISS: Injury Severity Score, RTS: Revised Trauma Score, TRISS: Trauma and Injury Severity Score; ^a^ includes 27,150 pedestrians and other vehicle-involved traffic-related injuries, neither of which are included in the Bicycle, Car, or Motorcycle categories; ^b^ analysed for drivers only; bold: highest value among vehicle categories.

## Data Availability

Bicycle-related casualty and mortality data (the Annual Report of Traffic Accidents released by the National Police Agency and vital statistics registration of the Ministry of Health, Labour, and Welfare in Japan) were derived from a source in the public domain and available at https://www.e-stat.go.jp. Restrictions apply to the availability of the demographic data and are not publicly available. Data were obtained from The Japan Trauma Data Bank (JTDB) database and are available with the permission of Japan Trauma Care and Research, which requires appropriate ethical and governance clearances.

## Supplementary Materials

The following supporting information can be downloaded at: https://www.mdpi.com/article/10.3390/ijerph22030322/s1, Figure S1: Proportion of bicycle-related casualties per population by age-group (2010–2020); Figure S2: Proportion of bicycle-related deaths per population by age-group (1995–2020); (Top) Men, (Bottom) Women; Figure S3: Proportion of deaths out of bicycle-related casualties by age-group (2010–2020); Table S1: Results of Poisson regression analysis of incidence rate ratios of bicycle-related casualties per population by age-group ((a) 2010, (b) 2020); Table S2: Results of Poisson regression analysis of IRR of bicycle-related deaths per population by age-group and year ((a) 1995–2009, (b) 2010–2020); Table S3: Number and percentage of injured body region by age-group (2004–2018).
