# Supplementary material for: Trends in and Risk Factors for Bicycle-Related Mortality in an Ageing Cycling-Centric Country: Analysis of Japanese Administrative Data
Source: Int J Environ Res Public Health. 2025 Feb 21;22(3):322. doi: 10.3390/ijerph22030322 (PMC11941933; doi:10.3390/ijerph22030322)
Supplement: Supplementary file 1 [file ijerph-22-00322-s001.zip › ijerph-3301998-supplementary correct version.pdf]

Supplementary Materials

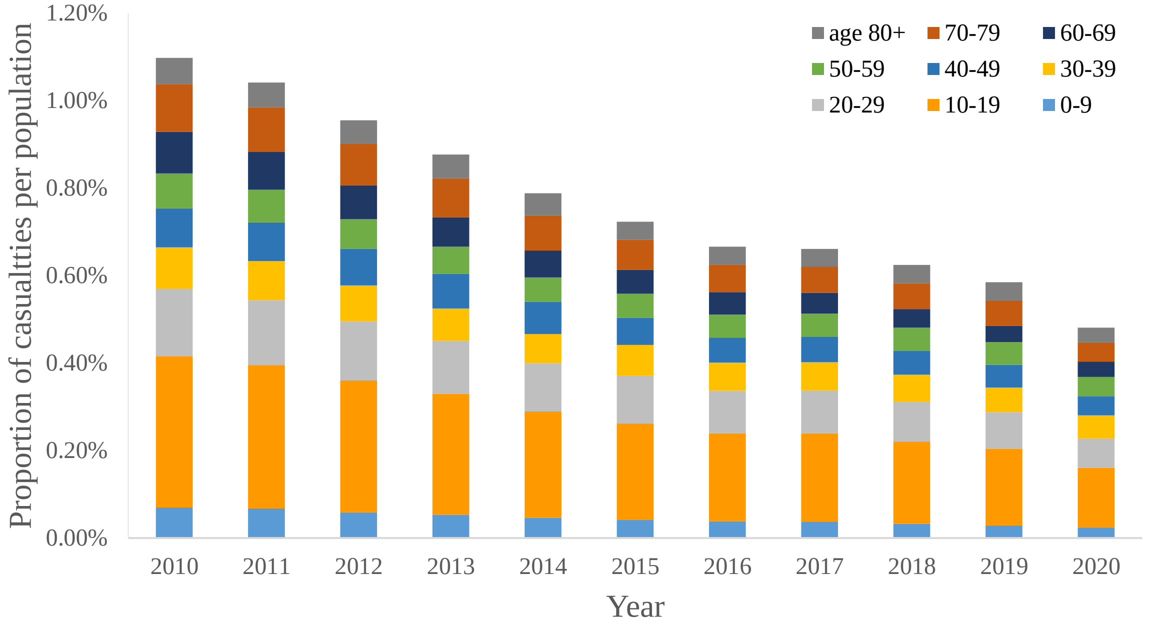

Figure S1. Proportion of bicycle-related casualties per population by age-group (2010-2020)

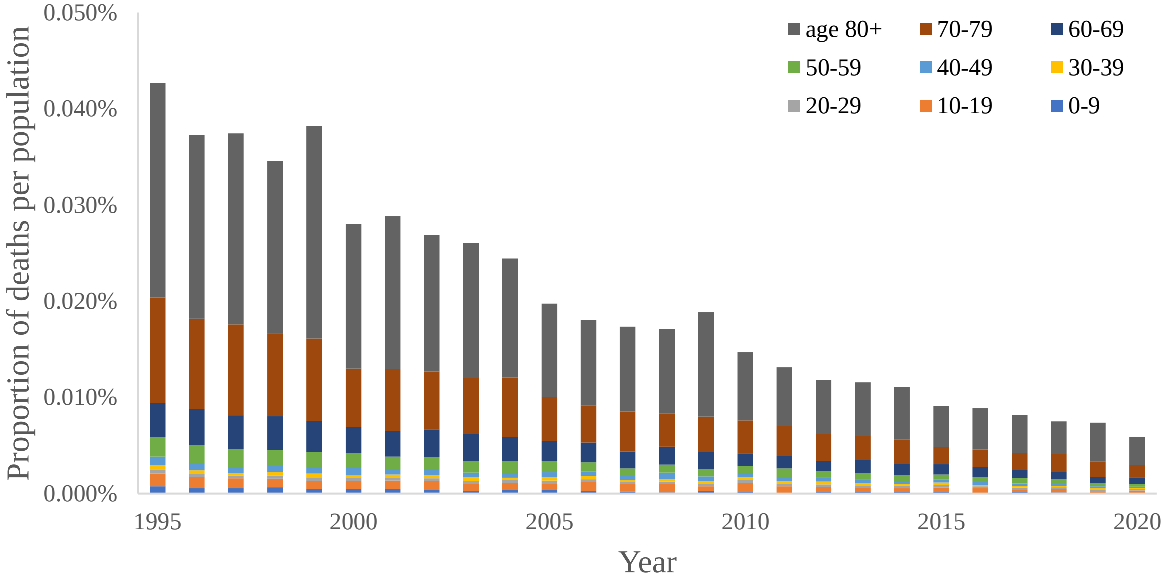

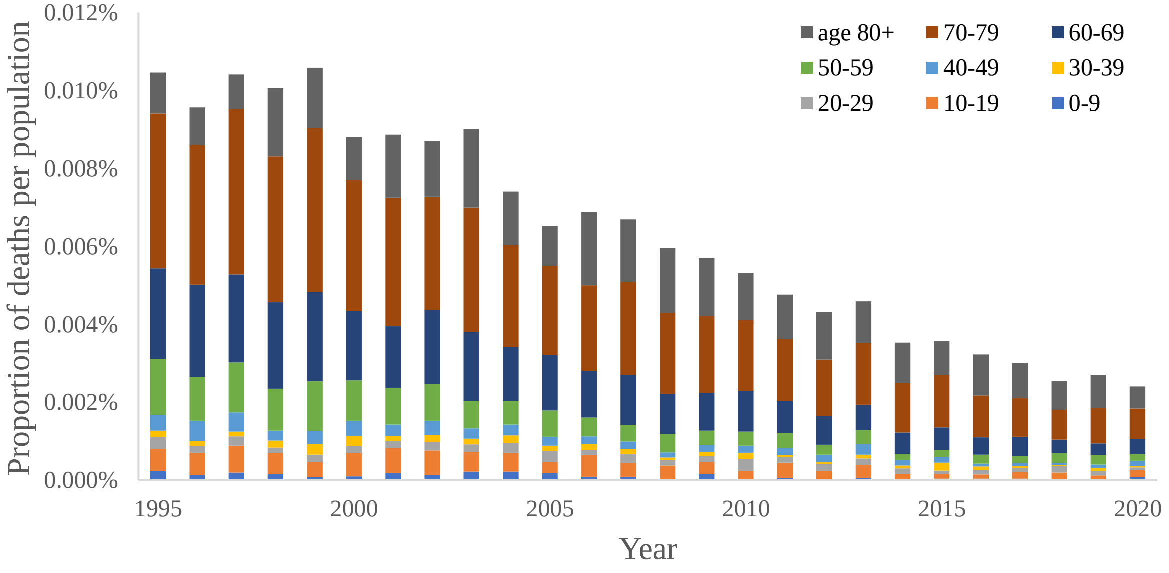

Figure S2. Proportion of bicycle-related deaths per population by age-group (1995-2020); Top) Men, Bottom) Women

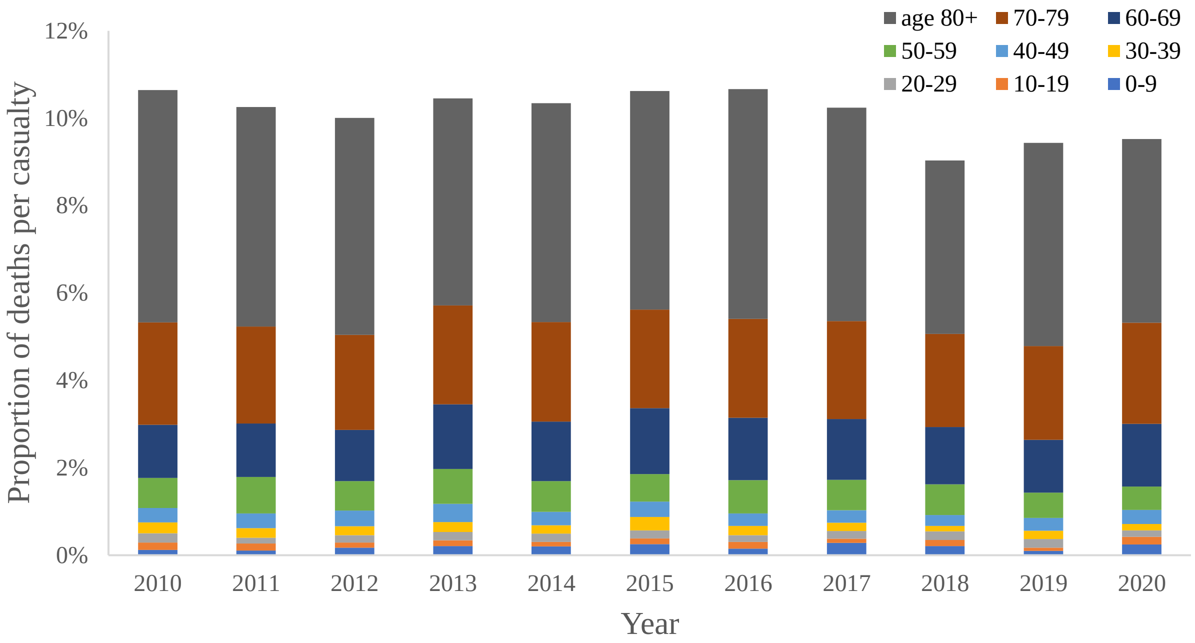

Figure S3. Proportion of deaths out of bicycle-related casualties by age-group (2010-2020)

Table S1. Results of Poisson regression analysis of incidence rate ratios of bicycle-related casualties per population by age-group ((a)2010, (b)2020)

(a)

	N (151,676)	Casualty			
		IRR	95% CI		*p*-value
			Lower	Upper	
**Age-group (years)**					
**0-9**	7,623	0.79	0.77	0.81	<0.01
**10-19**	41,476	3.89	3.82	3.96	<0.01
**20-29**	21,006	1.72	1.68	1.76	<0.01
**30-39**	17,295	1.10	1.05	1.10	<0.01
**40-49**	14,931	1 (reference)			
**50-59**	12,988	0.90	0.87	0.92	<0.01
**60-69**	17,423	1.07	1.05	1.10	<0.01
**70-79**	14,060	1.22	1.20	1.25	<0.01
**80+**	4,874	0.67	0.65	0.70	<0.01

(b)

	N (66,137)	Casualty			
		IRR	95% CI		*p*-value
			Lower	Upper	
**Age-group (years)**					
**0-9**	2,437	0.55	0.53	0.57	<0.01
**10-19**	15,452	3.08	3.00	3.17	<0.01
**20-29**	8,655	1.52	1.48	1.57	<0.01
**30-39**	7,729	1.19	1.16	1.23	<0.01
**40-49**	8,281	1 (reference)			
**50-59**	7,162	0.99	0.96	1.02	<0.01
**60-69**	5,650	0.80	0.77	0.83	<0.01
**70-79**	7,037	1.00	0.97	1.03	<0.01
**80+**	3,734	0.77	0.74	0.80	<0.01

Table S2. Results of Poisson regression analysis of IRR of bicycle-related deaths per population by age-group and year ((a) 1995-2009, (b) 2010-2020)

(a)

	Boys/Men				Girls/Women			
	IRR	95% CI		*p*-value	IRR	95% CI		*p*-value
		Lower	Upper			Lower	Upper	
**Year**	**0.96**	**0.95**	**0.98**	**<0.01**	**0.93**	**0.91**	**0.95**	**<0.01**
**Age-group (years)**								
**0-9**	0.89	0.73	1.09	0.27	0.38	0.27	0.53	<0.01
**10-19**	1.46	1.24	1.71	<0.01	1.32	1.06	1.64	0.01
**20-29**	0.46	0.37	0.56	<0.01	0.47	0.36	0.61	<0.01
**30-39**	0.51	0.41	0.63	<0.01	0.41	0.30	0.54	<0.01
**40-49**	1 (reference)				1 (reference)			
**50-59**	2.62	2.27	3.02	<0.01	3.09	2.57	3.72	<0.01
**60-69**	4.93	4.31	5.64	<0.01	5.32	4.46	6.30	<0.01
**70-79**	13.63	11.96	15.53	<0.01	9.04	7.60	10.76	<0.01
**80+**	28.86	25.29	32.94	<0.01	2.45	1.96	3.07	<0.01
**Age-group/year interaction**								
**0-9**	0.95	0.92	0.98	<0.01	1.04	1.00	1.01	0.82
**10-19**	1.00	0.97	1.02	0.65	1.04	1.01	1.07	<0.05
**20-29**	1.01	0.98	1.04	0.39	1.06	1.02	1.10	<0.05
**30-39**	1.01	0.98	1.04	0.59	1.04	1.00	1.09	<0.05
**40-49**	1 (reference)				1 (reference)			
**50-59**	0.97	0.95	0.99	<0.05	0.99	0.96	1.01	0.30
**60-69**	0.98	0.96	1.00	<0.05	1.01	0.99	1.04	0.35
**70-79**	0.96	0.94	0.97	<0.01	1.02	1.00	1.05	0.11
**80+**	0.97	0.95	0.99	<0.01	1.11	1.01	1.14	<0.01

CI: confidence interval, IRR: incidence rate ratio

(b)

	Boys/Men				Girls/Women			
	IRR	95% CI Lower	95% CI Upper	*p*-value	IRR	95% CI Lower	95% CI Upper	*p*-value
**Year**	**0.92**	**0.88**	**0.95**	**<0.01**	**0.91**	**0.87**	**0.97**	**<0.01**
**Age-group (years)**								
**0-9**	0.28	0.56	1.42	0.13	0.03	0.02	0.54	<0.05
**10-19**	3.18	1.14	8.84	<0.05	2.53	0.53	12.03	0.25
**20-29**	0.45	0.14	1.45	0.18	1.39	0.28	6.96	0.69
**30-39**	1.00	0.32	3.11	1.00	0.11	0.02	0.58	<0.01
**40-49**	1 (reference)				1 (reference)			
**50-59**	1.73	0.70	4.27	0.24	3.67	0.92	14.61	0.07
**60-69**	2.48	1.09	5.66	<0.05	14.72	4.51	48.04	<0.01
**70-79**	7.56	3.44	16.61	<0.01	21.61	6.99	66.85	<0.01
**80+**	12.80	5.85	28.02	<0.01	11.15	3.44	36.13	<0.01
**Age-group/year interaction**								
**0-9**	1.02	0.94	1.11	0.66	1.12	0.97	1.30	0.12
**10-19**	0.96	0.91	1.01	0.13	0.96	0.98	1.05	0.38
**20-29**	1.03	0.97	1.10	0.31	0.98	1.02	1.07	0.68
**30-39**	0.98	0.93	1.04	0.54	1.10	1.01	1.12	0.03
**40-49**	1 (reference)				1 (reference)			
**50-59**	1.01	0.96	1.05	0.81	0.95	0.95	1.03	0.20
**60-69**	1.02	0.97	1.06	0.45	0.93	0.97	0.99	<0.05
**70-79**	1.00	0.96	1.04	0.97	0.95	0.98	1.00	0.06
**80+**	1.01	0.97	1.05	0.57	0.97	1.01	1.03	0.28

CI: confidence interval, IRR: incidence rate ratio

Table S3. Number and percentage of injured body region by age-group (2004-2018)

	Age-group (years)								
Region	0-9	10-19	20-21	30-39	40-49	50-59	60-69	70-79	80+
Head	N= 748<br>(55.99%)	2384<br>(**58.59**)	956<br>(53.95)	794<br>(47.66)	1037<br>(49.52)	1246<br>(49.64)	2093<br>(53.15)	2393<br>(**57.41**)	1476<br>(**56.83**)
Face	71<br>(5.31)	259<br>(6.37)	166<br>(**9.37**)	121<br>(7.26)	124<br>(5.92)	107<br>(4.26)	125<br>(3.17)	102<br>(2.45)	52<br>(2.00)
Neck	1<br>(0.07)	6<br>(0.15)	3<br>(0.17)	5<br>(**0.30**)	6<br>(0.29)	4<br>(0.16)	7<br>(0.18)	1<br>(0.02)	2<br>(0.08)
Thorax	77<br>(5.76)	268<br>(6.59)	165<br>(9.31)	202<br>(12.12)	267<br>(12.75)	355<br>(**14.14**)	405<br>(10.28)	399<br>(9.57)	240<br>(9.24)
Abdomen	100<br>(**7.49**)	226<br>(5.55)	69<br>(3.89)	56<br>(3.36)	48<br>(2.29)	37<br>(1.47)	59<br>(1.50)	59<br>(1.42)	31<br>(1.19)
Spine	16<br>(1.20)	186<br>(4.57)	179<br>(10.10)	222<br>(13.33)	302<br>(14.42)	364<br>(14.50)	595<br>(**15.11**)	411<br>(9.86)	231<br>(8.89)
Upper Extremity	156<br>(**11.68**)	333<br>(8.18)	95<br>(5.36)	89<br>(5.34)	105<br>(5.01)	114<br>(4.54)	163<br>(4.14)	161<br>(3.86)	98<br>(3.77)
Lower Extremity	167<br>(12.50)	407<br>(10.00)	139<br>(7.84)	177<br>(10.62)	205<br>(9.79)	283<br>(11.27)	491<br>(12.47)	642<br>(15.40)	467<br>(**17.98**)

Bold: Age-group with highest percentage (top 3 for head)
